# Translating genomic medicine to the clinic: challenges and opportunities

**DOI:** 10.1186/s13073-019-0622-1

**Published:** 2019-02-22

**Authors:** Huan Zhang, Lars Klareskog, Andreas Matussek, Stefan M. Pfister, Mikael Benson

**Affiliations:** 1 Centre for Personalised Medicine, Linköping University, Linköping, Sweden; 2 Crown Princess Victoria Children’s Hospital, Linköping, Sweden; 3 Rheumatology Unit, Department of Medicine, Karolinska Institutet, Karolinska University Hospital (Solna), 171 76 Stockholm, Sweden; 4 Karolinska University Hospital Laboratory, Stockholm, Sweden; 5 Hopp Children’s Cancer Center Heidelberg (KiTZ), Heidelberg, Germany; 6 German Cancer Research Center (DKFZ), Heidelberg, Germany; 7 German Cancer Consortium (DKTK), Partner Site Heidelberg, Heidelberg, Germany; 8 Department of Pediatric Oncology, Hematology and Immunology, Heidelberg University Hospital, Heidelberg, Germany

## Abstract

Genomic medicine has considerable potential to provide novel diagnostic and therapeutic solutions for patients who have molecularly complex diseases and who are not responding to existing therapies. To bridge the gap between genomic medicine and clinical practice, integration of various data types, resources, and joint international initiatives will be required.

## Toward precise genomic therapies

From both public health and economic perspectives, lack of treatment response is one of the most pressing health care concerns that exist today. According to a US Food and Drug Administration (FDA) report from 2013, an astounding 38–75% of patients who have a common disease do not respond to treatment. Addressing this problem will be particularly important in increasing quality of life, decreasing mortality, and in turn, reducing health care costs globally. As an example, a 10% reduction in the number of patients who are not responding adequately to treatments for diabetes and heart disease in the USA would save approximately $200 billion annually [1].

Fundamental to tackling the issue of differential treatment response is the molecular complexity of common diseases, the scale of which can be vast; a disease may involve thousands of genes across multiple cell types in different parts of the body. In general, tools for clinical diagnostics do not allow for the assessment of such broad-scale changes. Thus, there is a wide gap between the molecular complexity and much of the translational research and clinical practice. Genomic medicine is emerging as a potential solution to bridge this gap [2]. Fundamental to this concept is the use of genome-wide analses to match precisely an individual patient’s molecular alterations with a drug targeting those alterations. This strategy is already being clinically implemented to treat rare monogenic diseases [3]. In cancer, the focus has mainly been on somatic genetic alterations. The development of increasingly sophisticated analyses of different molecular layers has, however, led to interest in other forms of omics analyses, such as analyses of the transcriptome, epigenome, or metabolome. These promising developments have led to several efforts aimed at implementing genomic medicine in clinical practice for early disease detection or molecularly tailored treatments. Examples include Obama’s Precision Medicine Initiative and several other international projects [2]. Nevertheless, concerns about the effectiveness of genomic medicine have led to questions about what genomic medicine has achieved to date, unresolved problems, and potential solutions.

Here, we discuss some clinically relevant examples and what can be learned from early attempts to implement genomic medicine in clinical practice. In summary, we propose that the clinical translation of genomic medicine will require the integration of multi-omics and clinical data and that emerging technologies such as single-cell approaches may facilitate the construction of high-resolution disease models. It is also possible that the complexity of diseases and technologies, coupled with the rapid speed of developments, will require the re-organization of health care structures in order to enable joint efforts to identify, evaluate, and implement new solutions for genomic medicine quickly.

## Promise and pitfalls of genomic medicine, focusing on genetic alterations

An early, successful example of a drug that targets somatic genetic alterations was imatinib, a tyrosine kinase inhibitor that is used for the treatment of chronic myeloid leukemia patients who have a translocation that results in the formation of a constitutively active BCR-ABL fusion kinase [2]. Other successful examples include the treatment of lung cancers harboring mutated epidermal growth factor receptor (EGFR) with EGFR inhibitors, and the treatment of melanomas bearing mutated BRAF with BRAF inhibitors [2].

These successful examples have spurred concerted efforts to generalize genomic approaches to the treatment of disease, including those based at the National Cancer Institute (NCI), the National Institutes of Health (NIH), and several European and Asian centers. An ideal outcome could be routine sequencing of all tumors so that actionable genetic alterations could be identified and prioritized for treatment, but the clinical relevance of this paradigm has been questioned. Currently, most cancer patients do not have actionable genetic alterations [2]. The SHIVA trial found no significant improvement in survival for cancer patients treated on the basis of actionable genetic alterations compared with those treated according to physician’s choice [4]. Possible explanations could be that those alterations did not have driving roles or that the patients also had other, untargeted alterations. Thus, from a gene-centered therapeutic perspective, the improved selection of targets or the use of combinatorial treatments is needed. It has also been argued, however, that the most effective current cancer treatments do not target mechanisms that are related to genetic alterations [2, 5]. This has led to increasing interest in including sources of information beyond genetic alterations.

## Multi-omics and multi-cellular approaches in the clinical translation of genomic medicine

In a recent study of brain tumors, genome-wide DNA methylation patterns were shown to agree with histopathological classification and led to the reclassification of some patients [6]. There are also examples of diagnostic transcriptomics for cancer and complex diseases [7]. It is reasonable to hypothesize that the integration of the most predictive markers from different omics layers will have greater potential than the assessment of genetic alterations alone [8]. In this context, we should mention that there are vast resources of multi-omics data in the public domain that can be mined for translational purposes [9].

Another important aspect is that most diseases involve multiple pathogenic mechanisms that are dispersed in multiple cell types, which may require combinatorial treatments. Several such examples are well established today. Asthma patients are commonly treated with inhalants that combine glucocorticoids and bronchodilators. In cancer, checkpoint inhibitors that increase immune responses complement drugs that directly target tumor cells. These examples highlight a potential limitation of many genomic analyses in finding diagnostic and therapeutic targets: the analyses are performed on bulk samples. Instead, we should ideally perform simultaneous genome-wide analyses of each individual disease-associated cell type, so that key regulatory cell types and mechanisms can be prioritized and targeted. The emergence of genome-wide methods for single-cell analyses has been proposed as a potential solution [7]. A large number of studies on cancer and complex diseases support this potential, and although there are currently multiple technical limitations, it is possible that rapid developments in single-cell analyses will result in high-resolution multicellular disease models for drug development and even diagnostics [7].

## Integration of omics and clinical data

Recent genomic and epigenomic studies of cancer have supported the diagnostic potential of integrating omics and histopathological data [6, 10]. There are now several examples of public and private efforts with similar aims that have acquired omics, clinical, and imaging data from hundreds of thousands of patients [2]. The DNA methylation study also presented a web-based diagnostic tool that can be used for standardized diagnostics in clinics across the globe [7]. Importantly, the use of this tool in clinical practice will result in the continuous improvement of the tool and potentially in an improved understanding of pathogenic mechanisms that result in disease, as well as of the diagnostics and therapeutics. The study may serve as a model for how different combinations of local or centralized wet lab and computational analyses may contribute significantly to the clinical implementation of genomic medicine.

## Challenges and opportunities for research and clinical practice

The diagnostic and therapeutic success stories mentioned above support the potential for translating genomic medicine to the clinic. Nevertheless, there is a wide gap between the complexity of the diseases and current clinical practice. Bridging that gap involves addressing several different challenges: from a research perspective, the clinical translation of genomic medicine requires the integration of multiple disciplines, such as omics, epidemiology, bioinformatics, and experimental and clinical research. Such integration is complicated by the compartmentalization of academic and industrial research. A related problem for funders and medical journals is the evaluation of multi-disciplinary research when most reviewers have expertise only in some of the disciplines involved. From a health care perspective, the rapid development of multiple solutions for genomic medicine is likely to make the identification, evaluation, and prioritization of “actionable” diagnostic and therapeutic solutions important challenges to be addressed urgently. Next, the clinical implementation of successful solutions may require new technologies as well as the training of health professionals. This could result in the development of novel clinical disciplines, derived from integrating genomics, systems medicine, and bioinformatics. Taken together, these challenges and opportunities may require new organizational solutions in academia and health care. As an example, the web-based analyses of DNA methylation data from brain cancers suggest that novel combinations of local and centralized solutions for diagnostics may become optimal. This could lead to new roles for health care units that might serve increasingly as dynamic integrators and providers of local and global services—as opposed to focusing on the local accumulation of expertise and technologies. Such a development would require new solutions for the continuous education of health professionals, as well as organizational flexibility to optimize the integration of resources.

## Conclusions

The huge medical, economic, and societal problems arising from the large number of patients who do not respond to therapy emphasize the need for novel diagnostic and therapeutic solutions for the management of patients. Genomic medicine has considerable potential, but also presents significant challenges that are likely to be addressed by joint international initiatives.
